# Analysis of the role of Frizzled 2 in different cancer types

**DOI:** 10.1002/2211-5463.13111

**Published:** 2021-02-25

**Authors:** Miaomiao Zhou, Xuezhu Sun, Yunhao Zhu

**Affiliations:** ^1^ West Anhui Health Vocational College Anhui China

**Keywords:** frizzled 2 receptor, pan‐cancer, tumor microenvironment, drug sensitivity

## Abstract

Frizzled 2 (*FZD2*) is an important receptor in the Wnt pathway, which is highly expressed in malignant tumors and helps regulate multiple tumor behaviors. Its expression level is related to prognosis. Here, bioinformatic analysis was performed to understand the expression of *FZD2* in different tumors. We examined *FZD2* expression using pan‐cancer data of 33 cancer types from The Cancer Genome Atlas (TCGA). Differential expression analysis (Wilcoxon's test) was used to compare tumor and normal tissues. Univariate Cox proportional hazard regression was performed to compare gene expression and overall patient survival. COSMIC, cBioPortal, and CCLE were used to examine *FZD2* mutations in human cancers. Dryness index was calculated using one‐class logistic regression (OCLR). Spearman's correlation was performed based on gene expression and dryness score and used to analyze the correlation between gene expression and stemness score, matrix score, immune score, estimated score, tumor mutation burden (TMB), microsatellite instability (MSI), and drug sensitivity. STRING website was used to construct an *FZD2* protein interaction network and identify genes that interact with *FZD2*. We report that *FZD2* is highly expressed in most tumors, differing between cancer types. Expression was related to patient overall survival (OS), disease‐specific survival, disease‐free interval (DFI), mutations, drug sensitivity, tumor microenvironment, immune cell infiltration, immune checkpoint gene expression, immunotherapy indicators (TMB, MSI), and tumor cell stemness. *FZD2* influenced drug sensitivities, including cobimetinib (*r* = −0.553, *P* < 0.001), selumetinib (*r* = −0.539, *P* < 0.001), bafetinib (*r* = −0.538, *P* < 0.001), tamoxifen (*r* = −0.523, *P* < 0.001), alvespimycin (*r* = −0.520, *P* < 0.001), and nilotinib (*r* = −0.502, *P* < 0.001). *FZD2* has the most significant correlation with *ROR2* (*r* = 0.4, *P* < 0.001), *Wnt2* (*r* = 0.37, *P* < 0.001), and *Wnt4A* (*r* = 0.34, *P* < 0.001). The results confirm the importance of *FZD2* expression in cancer prognosis and treatment, and provide new clues for treatment strategies.

## Graphical abstract

The expression of FZD2 is related to patient overall survival (OS), disease‐specific survival, disease‐free interval (DFI), mutations, drug sensitivity, tumor microenvironment, immune cell infiltration, immune checkpoint gene expression, immunotherapy indicators (TMB, MSI), and tumor cell stemness. The high expression level of FZD2 is significantly related to Wnt signaling pathway.

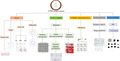

**Abbreviations**
ACC: Adrenocortical carcinoma
BLCA: Bladder urothelial carcinoma
BRCA: Breast invasive carcinoma
CCLE: Cancer Cell Line Encyclopedia
CESC: Cervical squamous cell carcinoma and endocervical adenocarcinoma
CHOL: Cholangiocarcinoma
COAD: Colon adenocarcinoma
COSMIC: The Catalog of Somatic Mutations in Cancer
CSCs: Cancer stem cells
DCs: Dendritic cells
DFI: Disease‐free interval
DLBC: Lymphoid neoplasm diffuse large B‐cell lymphoma
DNAss: Dryness index based on DNA methylation
DSS: Disease‐specific survival
ESCA: Esophageal carcinoma
FZD2: Frizzled 2
FZDs: Frizzled
GBM: Glioblastoma multiforme
GC: Gastric cancer
HNSC: Head and neck squamous cell carcinoma
KICH: Kidney chromophobe
KIRC: Kidney renal clear cell carcinoma
KIRP: Kidney renal papillary cell carcinoma
LAML: Acute myeloid leukemia
LGG: Brain lower‐grade glioma
LIHC: Liver hepatocellular carcinoma
LUAD: Lung adenocarcinoma
LUSC: Lung squamous cell carcinoma
MESO: Mesothelioma
MSI: Microsatellite instability
NCI: National Cancer Institute
OCLR: One‐class logistic regression
OS: Overall survival
OV: Ovarian serous cystadenocarcinoma
PAAD: Pancreatic adenocarcinoma
PCPG: Pheochromocytoma and paraganglioma
PPI: Protein–protein interaction
PRAD: Prostate adenocarcinoma
READ: Rectum adenocarcinoma
RNAss: Dryness index based on mRNA expression
SARC: Sarcoma
SKCM: Skin cutaneous melanoma
STAD: Stomach adenocarcinoma
STRING: The Search Tool for the Retrieval of Interacting Genes/Proteins
TCGA: The Cancer Genome Atlas
TGCT: Testicular germ cell tumors
THCA: Thyroid carcinoma
THYM: Thymoma
TMB: Tumor mutation burden
UCEC: Uterine corpus endometrial carcinoma
UCS: Uterine carcinosarcoma
UVM: Uveal melanoma

Frizzled receptors (*FZDs*) are seven‐span membrane proteins belonging to a subclass of the G protein‐coupled receptor family [1]. There are 10 *FZDs* in human cells (*FZD1*‐*FZD10*) [1]. There are 19 members of the Wnt family that can bind to these 10 members of the *FZD* family to activate the Wnt/β‐catenin pathway [2]. Abnormal activation of the Wnt pathway plays an important role in cell carcinogenesis, tumorigenesis, and invasion. Abnormally activation of Wnt through its signaling pathway may cause tumors [3]. *FZD* is usually affected in these cases, and abnormal expression can be seen in a variety of malignant tumors; therefore, inhibiting this pathway may engender new breakthroughs in the treatment of tumors [4].

Frizzled 2 (*FZD2*) is a newly discovered tumor marker. It is one of the important receptors of the Wnt signaling pathway and is mainly involved in nonclassical pathway signal transduction [5]. It is highly expressed in a variety of malignant tumors and participates in the regulation of various tumor behaviors [6, 7, 8, 9]. Its expression level is closely related to patient prognosis, and therefore, it is expected to become a new prognostic indicator and therapeutic target for a variety of cancers [10, 11]. Here, bioinformatic analysis was performed to understand the expression of *FZD2* in different tumors and its possible connection with cancer. This study used TCGA data to conduct a comprehensive analysis of *FZD2* expression characteristics, prognostic value, correlation of tumor‐infiltrating immune cells, and drug sensitivity, to provide more information to better understand the importance of *FZD2* in pan‐cancer.

## Materials and methods

### TCGA pan‐cancer data

On March 23, 2020, data on different types of cancer were downloaded from the Xena Browser (https://xenabrowser.net/datapages/), including gene expression RNA‐Seq (HTSeq‐FPKM), clinical data, and survival data. The pan‐cancer data of 33 primary tumors are described in Table 1.

**Table 1:** Pan‐cancer data of 33 primary from TCGA database.

TCGA ID	Cancer	Normal	Tumor
ACC	Adrenocortical carcinoma	0	79
BLCA	Bladder urothelial carcinoma	19	411
BRCA	Breast invasive carcinoma	120	1097
CESC	Cervical squamous cell carcinoma and endocervical adenocarcinoma	3	306
CHOL	Cholangiocarcinoma	9	36
COAD	Colon adenocarcinoma	41	471
DLBC	Lymphoid neoplasm diffuse large B‐cell lymphoma	0	48
ESCA	Esophageal carcinoma	11	162
GBM	Glioblastoma multiforme	5	168
HNSC	Head and neck squamous cell carcinoma	44	502
KICH	Kidney chromophobe	24	65
KIRC	Kidney renal clear cell carcinoma	72	535
KIRP	Kidney renal papillary cell carcinoma	32	289
LAML	Acute myeloid leukemia	0	152
LGG	Brain lower‐grade glioma	0	529
LIHC	Liver hepatocellular carcinoma	50	374
LUAD	Lung adenocarcinoma	59	526
LUSC	Lung squamous cell carcinoma	49	501
MESO	Mesothelioma	0	86
OV	Ovarian serous cystadenocarcinoma	0	379
PAAD	Pancreatic adenocarcinoma	4	178
PCPG	Pheochromocytoma and paraganglioma	3	183
PRAD	Prostate adenocarcinoma	52	499
READ	Rectum adenocarcinoma	10	167
SARC	Sarcoma	2	263
SKCM	Skin cutaneous melanoma	1	471
STAD	Stomach adenocarcinoma	32	375
TGCT	Testicular germ cell tumors	0	156
THCA	Thyroid carcinoma	58	510
THYM	Thymoma	2	119
UCEC	Uterine corpus endometrial carcinoma	35	548
UCS	Uterine carcinosarcoma	0	56
UVM	Uveal melanoma	0	80
Total		737	10 321

### Differential expression analysis of FZD*2* between normal and tumor samples

For all TCGA tumor types, the ‘ggpubr’ R software package was used to perform differential expression analysis (Wilcoxon's test) between tumor and normal tissues. Only tumor types with more than five normal samples were included. In the heat map, the difference in *FZD2* gene expression in pan‐carcinoma is presented in the form of log_2_ fold change (log_2_ FC).

### Clinical correlation analysis

The correlation between high and low levels of *FZD2* expression and overall survival (OS), disease‐specific survival (DSS), and disease‐free interval (DFI) was analyzed using an R software package (Kaplan–Meier diagram) using phenotype and survival data of 33 TCGA cancers from the GDC TCGA collection in the UCSC Xena database (http://xena.ucsc.edu/). According to the median expression level of *FZD2*, these were divided into high expression and low expression groups. In addition, Cox proportional hazard regression analysis was used to obtain the hazard ratio of *FZD2* in each TCGA tumor type. Furthermore, the differential analysis was used to detect differences in *FZD2* expression characteristic levels at different stages of the 33 cancers. *P* < 0.05 was considered statistically significant.

### Mutation analysis

The catalog of somatic mutations in cancer (COSMIC) database (https://cancer.sanger.ac.uk/cosmic/) collects millions of coding mutations, noncoding mutations, genome rearrangements, fusion genes, copy number abnormalities, and gene expression variations in the human genome [12]. In this study, COSMIC was used to examine *FZD2* mutations in human cancers. cBioPortal (http://cbioportal.org) is an open resource that can be used to interactively explore multiple sets of cancer genomic data [13]. In this study, cBioPortal was used to analyze the mutation rate and distribution of *FZD2* in different exons in TCGA pan‐cancer data. The Cancer Cell Line Encyclopedia (CCLE) project dataset is a compilation of gene expression data from human cancer cell lines and was used to analyze *FZD2* mutations in various cancer cell lines [14].

### Correlation analysis between tumor mutation burden and microsatellite instability

Tumor mutation burden (TMB) is defined as the total number of somatic gene coding errors, base substitutions, insertions, or deletions detected per million bases. The correlation between tumor mutation load and *FZD2* gene expression was calculated using Spearman's test; this was also used to calculate the correlation between microsatellite instability and *FZD2* expression. The result was represented by the R ‘fmsb’ package radar chart (***P < 0. 001; **P < 0. 01; *P < 0.05).

### TIMER

TIMER (https://cistrome.shinyapps.io/timer) provides cancer researchers with a comprehensive analysis network tool for analyzing immune cell infiltration in a variety of cancers [15]. The database uses statistical methods validated by pathological examinations to evaluate the immune infiltration of B cells, CD4+ T cells, CD8+ T cells, neutrophils, macrophages, and dendritic cells (DCs) on tumors. This database was used to analyze the correlation between *FZD2* expression and a large number of immune infiltrations.

### Stemness indices and tumor microenvironment in pan‐cancer

The tumor microenvironment mainly includes tumor cells, mesenchymal cells, and the extracellular matrix. These play an important role in tumor growth, angiogenesis, tumor invasion, and metastasis [16]. The ESTIMATE method was used to analyze the correlation between *FZD2* expression in TCGA tumor samples and the ratio of stromal cells and immune cells [17]. The ESTIMATE score is calculated based on gene expression characteristics, which can reflect the purity of the tumor; it also has good prediction accuracy. By using the estimation package and the limma package, a Spearman correlation analysis was performed between the expression level of *FZD2* and the matrix score.

To further analyze the relationship between *FZD2* and pan‐cancer stemness, a one‐class logistic regression (OCLR) machine learning algorithm was used to calculate the stemness index of TCGA tumor samples, and Spearman's correlation was performed based on gene expression and stemness score analysis [18]. The dryness indices based on DNA methylation (DNAss) and on mRNA expression (RNAss) were obtained.

### Analysis of drug sensitivity in pan‐cancer

The cancer cell line platform established by the National Cancer Institute (NCI) has been widely used in drug screening based on related gene expression. NCI‐60 is a collection of 60 human cancer cell lines from nine different cancer types (leukemia, colon cancer, lung cancer, cancers of the central nervous system, kidney cancer, melanoma, ovarian cancer, breast cancer, and prostate cancer). NCI‐60 expression data were obtained from CellMiner. The Pearson correlation coefficient was calculated to analyze the relationship between mRNA expression and the 50% growth inhibitory concentration of the drug.

### Establishment of protein–protein interaction (PPI) network

The Search Tool for the Retrieval of Interacting Genes/Proteins (STRING) website was used to construct the *FZD2* protein interaction network and obtain the genes that are mainly related to *FZD2*. TCGA was used for correlation analysis of genes related to *FZD2*.

## Results

### FZD2 gene expression in human cancers

**Fig. 1:**
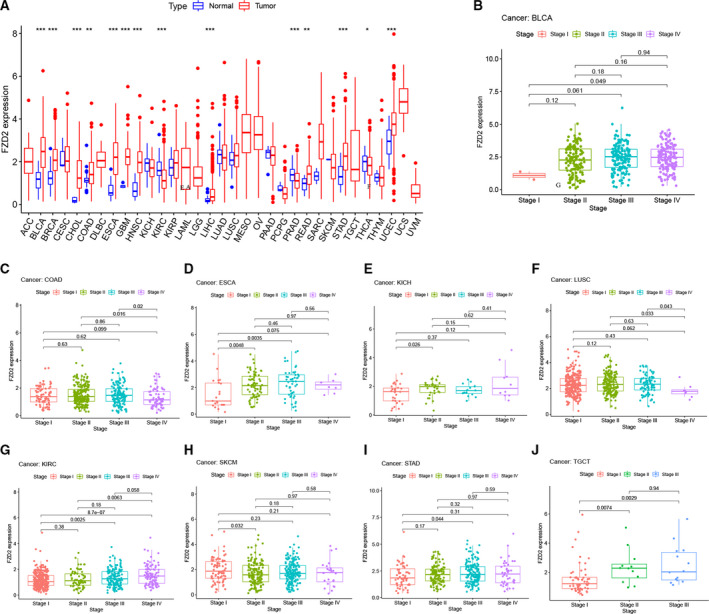
The mRNA expression of *FZD2* in pan‐cancer. (A) The mRNA expression of *FZD2* between tumor and normal tissues was assessed using tissues from TCGA (we used the Wilcoxon test for statistical analysis, and *P* < 0.05 was considered statistically significant). (B‐J) Correlation between *FZD2* mRNA expression and pathological stages in patients with BLCA, COAD, ESCA, KICH, LUSC, KIRC, SKCM, STAD, and TGCT. *P* < 0.05 was considered significant.

Tumor samples from the TCGA database were integrated to analyze *FZD2* mRNA expression characteristics. When only tumors in the TCGA and adjacent tissues were included, *FZD2* was found to be upregulated in BLCA, BRCA, CHOL, COAD, ESCA, GBM, HNSC, LIHC, READ, STAD, and UCES cancers (Fig. 1A). In the different clinical stages of BLCA, COAD, ESCA, KICH, KIRC, LUSC, SKCM, STAD, and TGCT, the mRNA expression of *FZD2* also differed significantly (Fig. 1B–J).

### Correlation analysis between FZD*2* expression level and prognosis

**Fig. 2:**
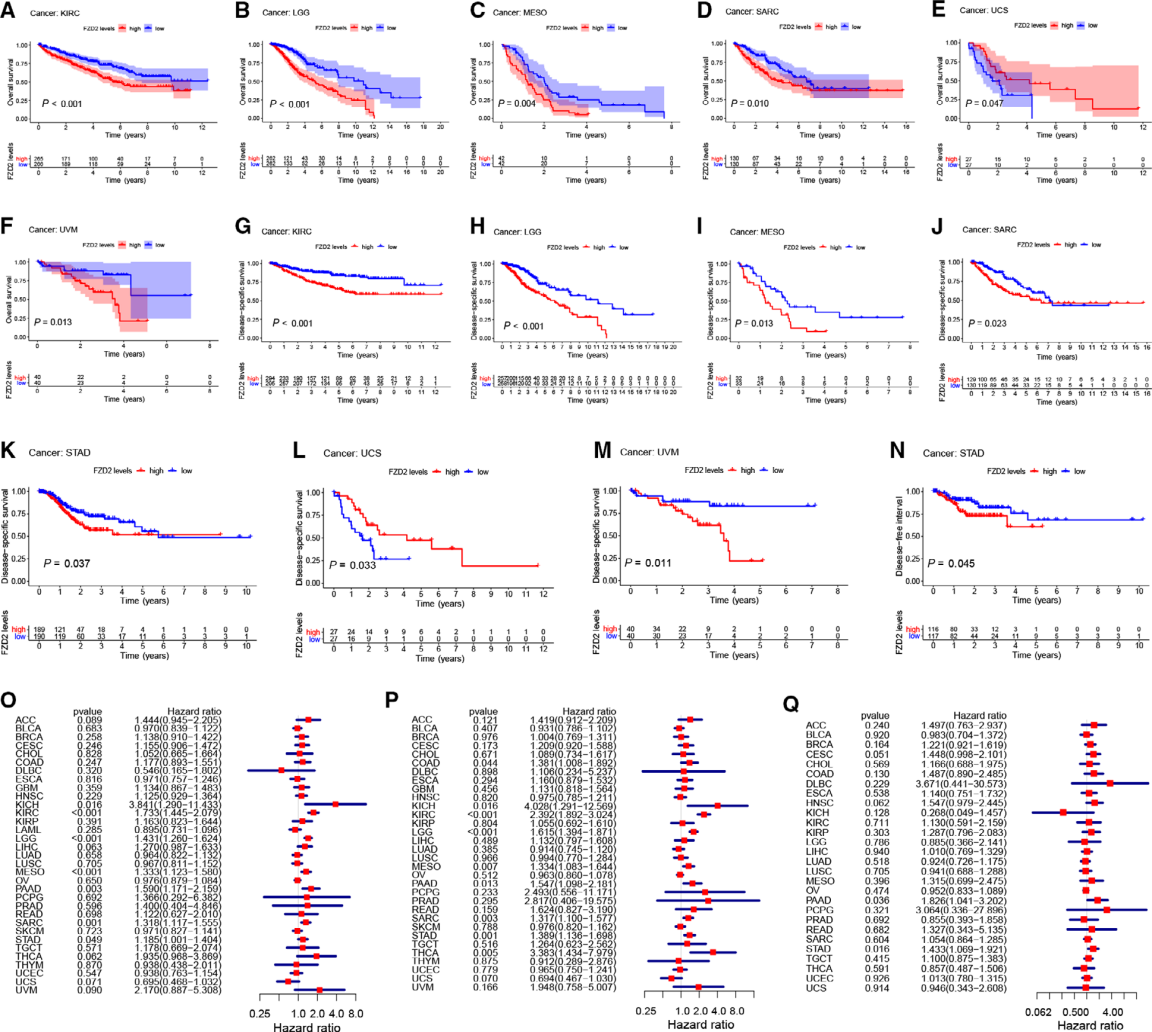
OS, DSS, and DFI difference between high and low *FZD2* mRNA expression groups in significant prognosis‐related tumors from TCGA database. (A–F) OS difference between groups in KIRC, LGG, MESO, SARC, UCS, and UVM. (G–M) DSS difference between groups in KIRC, LGG, MESO, SARC, STAD, UCS, and UVM. (N) DFI difference between groups in STAD. (O) Univariate Cox regression analysis was used to analyze the correlation between *FZD2* mRNA expression and OS. (P) Univariate Cox regression analysis was used to analyze the correlation between *FZD2* mRNA expression and DSS. (Q) Univariate Cox regression analysis was used to analyze the correlation between *FZD2* mRNA expression and DFI. *P* < 0.05 was considered significant.

Using data from the TCGA database, univariate Cox regression analysis was used to evaluate the correlation between *FZD2* mRNA expression levels and OS and DSS in different types of cancer. When the median expression value of each cancer type was classified, it was found that upregulation of *FZD2* expression was related to shorter OS and DSS in KIRC, LGG, MESO, SARC, and UVM. In contrast, upregulation of *FZD2* expression was related to the longer OS and DSS in UCS (Fig. 2A–M). The hazard ratios for *FZD2* were significant for KICH, KIRC, LGG, MESO, PAAD, SARC, and STAD, among which *FZD2* had the highest risk effect in KICH (Fig. 2O–P). The correlation between *FZD2* expression and DFI was analyzed using Cox regression, and a significant hazard ratio was found for STAD (Fig. 2N). According to the median expression of *FZD2* across the different cancer types, patients were divided into either a high or low expression group; when analyzed, it was found that the survival difference between the high and low expression groups was significant and that patients with high *FZD2* expression had earlier recurrence after tumor resection (Fig. 2Q).

### FZD2 mutations in pan‐cancer

**Fig. 3:**
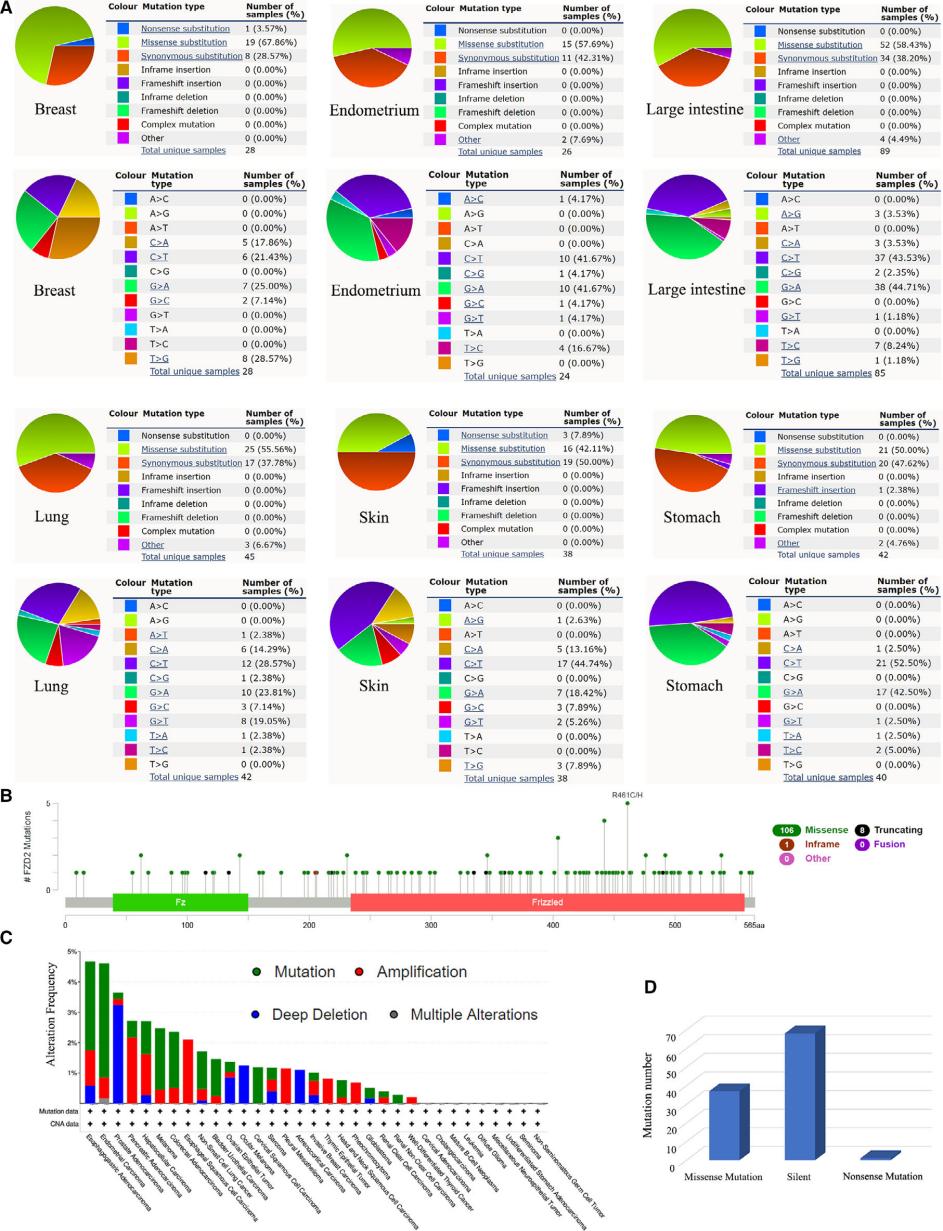
The alteration of *FZD2* in different cancers. (A) Pie chart showing the percentage of the different mutation types of *FZD2* in human cancers according to the COSMIC database. (B) Mutation diagram of *FZD2* in different cancer types across protein domains. (C) *FZD2* mutation level in the TCGA cancer database. (D) Mutation of *FZD2* in cancer cell lines obtained from the CCLE.

COSMIC provides information about *FZD2* mutations in different cancers, including missense mutations, nonsense mutations, and synonymous mutations (Figs 3A and Fig. S1). Synergistic mutations were obvious in breast cancer, endometrial cancer, large intestine cancer, liver cancer, lung cancer, skin cancer, and stomach cancer, while nonsense mutations were rare (Fig. 3A). The sample size of other tumor mutations was small, and different types of mutations also appeared (Fig. S1). C>T and G>A mutations were found to be the most common in the *FZD2* coding chain, while A>T and T>A mutations were rare. Fig. 3B,C shows the mutation result of cBioPortal, illustrating the mutation level of *FZD2* in the TCGA cancer database. A total of 106 mutation sites were found in *FZD2* through the cBioPortal database, located between amino acids 0 and 565 (Fig. 3B). Among these, the mutation rate was higher in esophagogastric adenocarcinoma and endometrial carcinoma (Fig. 3C). Missense mutations and silent mutations were also found in cancer cell lines (Fig. 3D).

### The relationship between FZD2 mRNA expression and tumor immune microenvironment

**Fig. 4:**
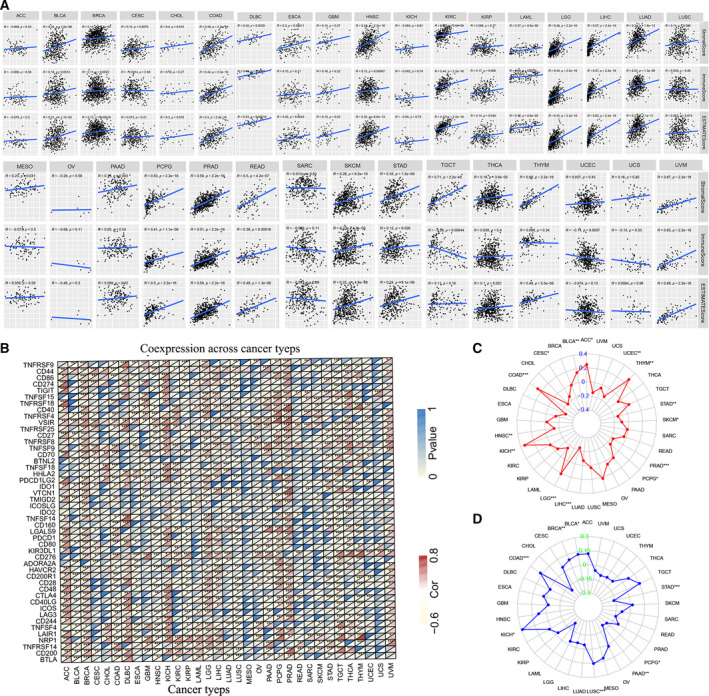
Relation between tumor microenvironment, TMB, MSI, immune checkpoints’ mRNA expression, and *FZD2* mRNA expression levels in various tumors in TCGA database. (A) The correlation between *FZD2* and stromal scores, immune scores, and ESTIMATE scores in pan‐cancer. Spearman's correlation tests were used for testing, and *P* < 0.05 was considered significant. (B) Correlation between *FZD2* mRNA expression levels and acknowledged immune checkpoints’ mRNA expression in multiple tumors from TCGA database. The lower triangle in each tile indicates coefficients calculated by Pearson's correlation test, and the upper triangle indicates log_10_‐transformed *P*‐value. *P < 0.05, **P < 0.01, ***P < 0.001. (C) Correlation between TMB and *FZD2* expression. Spearman's correlation test was used for testing, *P* < 0.05 was considered significant. (D) Correlation between MSI and *FZD2* expression. Spearman's correlation test was used for testing, *P* < 0.05 was considered significant.

After determining the prognostic value of *FZD2*, the relationship between *FZD2* and tumor‐infiltrating immune cells in cancer was explored. The ESTIMATE method was used to analyze the correlation between *FZD2* expression in TCGA tumor samples and the ratio of both stromal cells and immune cells (Fig. 4A). In COAD, DLBC, LGG, LIHC, PCPG, PRAD, READ, and UVM, it was found that *FZD2* significantly positively correlated with stromal score, immune score, and estimated score. *FZD2* had the highest correlation with stromal score in TCGT (*r* = 0.71, *P* < 0.001), while the highest correlation with immune score (*r* = 0.65, *P* < 0.001) and estimate score (*r* = 0.68, *P* < 0.001) was found in UVM. *FZD2* expression and immune cell infiltration were also analyzed using the TIMER database correlation between levels, where the expression of *FZD2* had the highest correlation with macrophages, DCs, and T‐cell CD4+ cells (Fig. S2).

### Correlation between FZD2 expression and certain immune checkpoint gene expression in certain cancers

Immune checkpoints are a class of inhibitory molecules that play a protective role in the human immune system, preventing excessive activation of T cells from causing damage to themselves. Tumor cells can exploit this protective mechanism by overexpressing the checkpoint molecules, inhibiting the antitumor response of the immune system to achieve immune escape. Immune checkpoint inhibitors act to block the interaction of immune checkpoints and their ligands, break immune tolerance, enhance immune cell activity, and promote immune clearance of tumor cells, thereby inhibiting the occurrence and development of tumors. The mRNA sequence database allows us to assess whether there is a link between *FZD2* expression and the expression of such checkpoint genes. The correlation analysis between *FZD2* and checkpoint gene expression revealed a high correlation in VISR, CD200, TNFRSF4, TNFRSF 14, NRP1, and CD44 in various types of cancer (*P* < 0.05). In addition, significant co‐expression of *FZD2* and other immune checkpoint genes was detected in Adrenocortical carcinoma (ACC), BRCA, DLBC, KICH, LGG, PCPG, PRAD, TGCT, and THYM. However, in TGCT and THYM, the expression of *FZD2* was negatively correlated with most immune checkpoint molecules (Fig. 4B).

### Relationship between FZD2 mRNA expression, and TMB and MSI in some cancers

The relationship between TMB and MSI and *FZD2* expression was examined in various cancer types. The results showed that the expression of *FZD2* correlated significantly with TMB in ACC, BLCA, CESC, COAD, HNSC, KICH, LGG, LIHC, PCPG, PRAD, STAD, SKCM, THYM, and UCEC (*P* < 0.05), and that KICH, COAD, and THYM had the highest coefficients, while LIHC had the lowest (Fig. 4C). The coefficient value indicates that *FZD2* expression was positively correlated with high mutation status in KICH, COAD, and THYM, and positively correlated with low mutation status in LIHC. The correlation between *FZD2* expression and MSI was analyzed in 33 cancers, and expression of *FZD2* was significantly correlated with MSI in BLCA, BRCA, COAD, KICH, LUSC, PAAD, PCPG, and STAD (*P* < 0.05; Fig. 4D). The coefficient of KICH was the highest, indicating a positive correlation between *FZD2* expression and MSI in this type. In contrast, the expression of *FZD2* had the lowest coefficients in PAAD, PCPG, and STAD, indicating that in these types there is a significant negative correlation between *FZD2* expression and MSI.

### Stemness indices in pan‐cancer

**Fig. 5:**
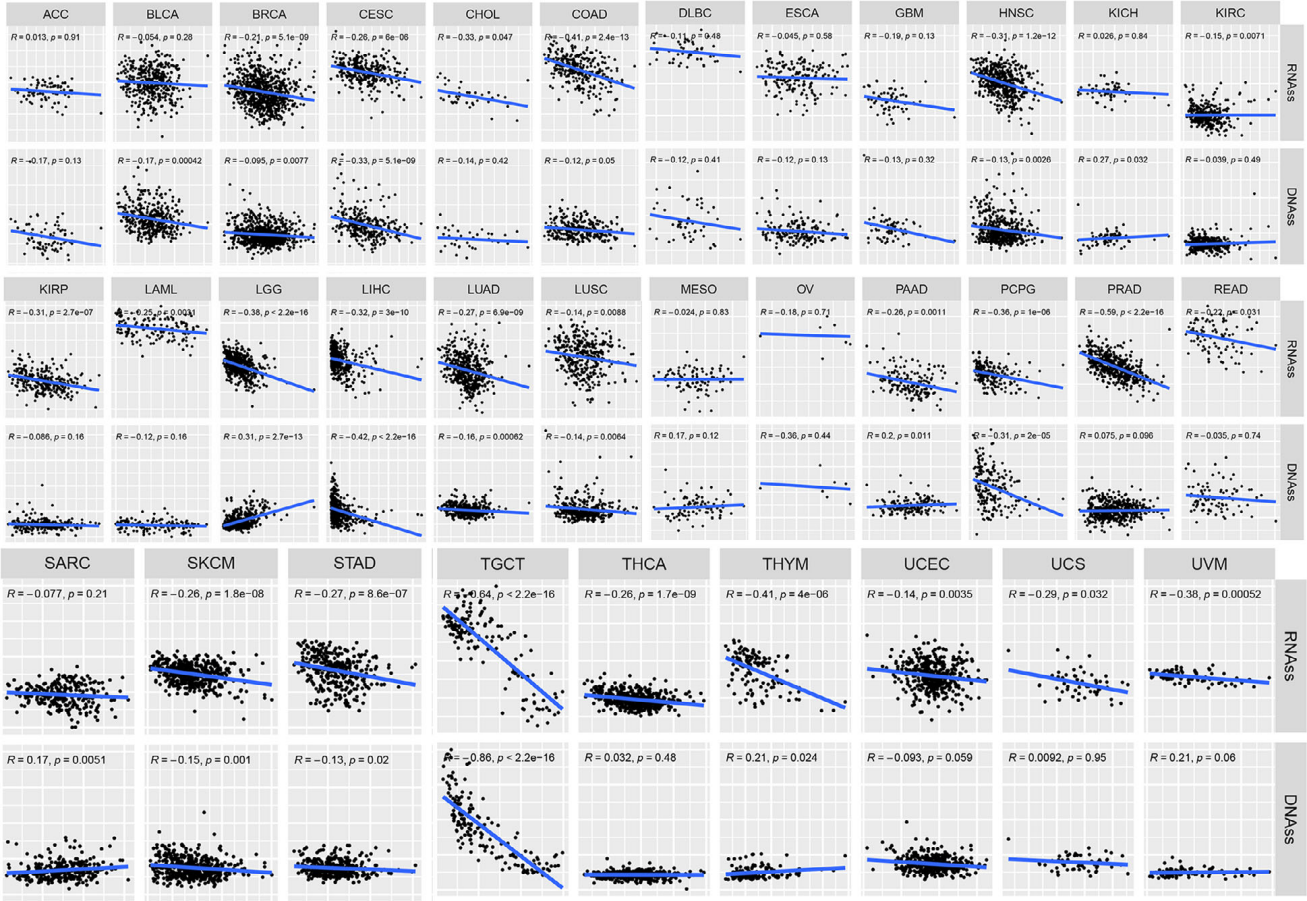
Correlation matrixes between *FZD2* expression and RNAss and DNAss. Spearman's correlation tests were used for testing, and *P* < 0.05 was considered significant.

The dryness index (DNAss) and the mRNA expression‐based dryness index (RNAss) were used to further understand the correlation between *FZD2* and dryness in pan‐cancer. In LGG, LIHC, PCPG, and TCGT, *FZD2* has a strong correlation with DNAss and RNAss. In DNAss, *FZD2* had a significant negative correlation with TCGT (*r* = −0.64, *P* < 0.001) and PRAD (*r* = −0.59, *P* < 0.001). For RNAss, there was a significant negative correlation between *FZD2* and TCGT (*r* = −0.86, *P* < 0.001) and LIHC (*r* = −0.42, *P* < 0.001; Fig. 5).

### Analysis of drug sensitivity in FZD2 and pan‐cancer

**Fig. 6:**
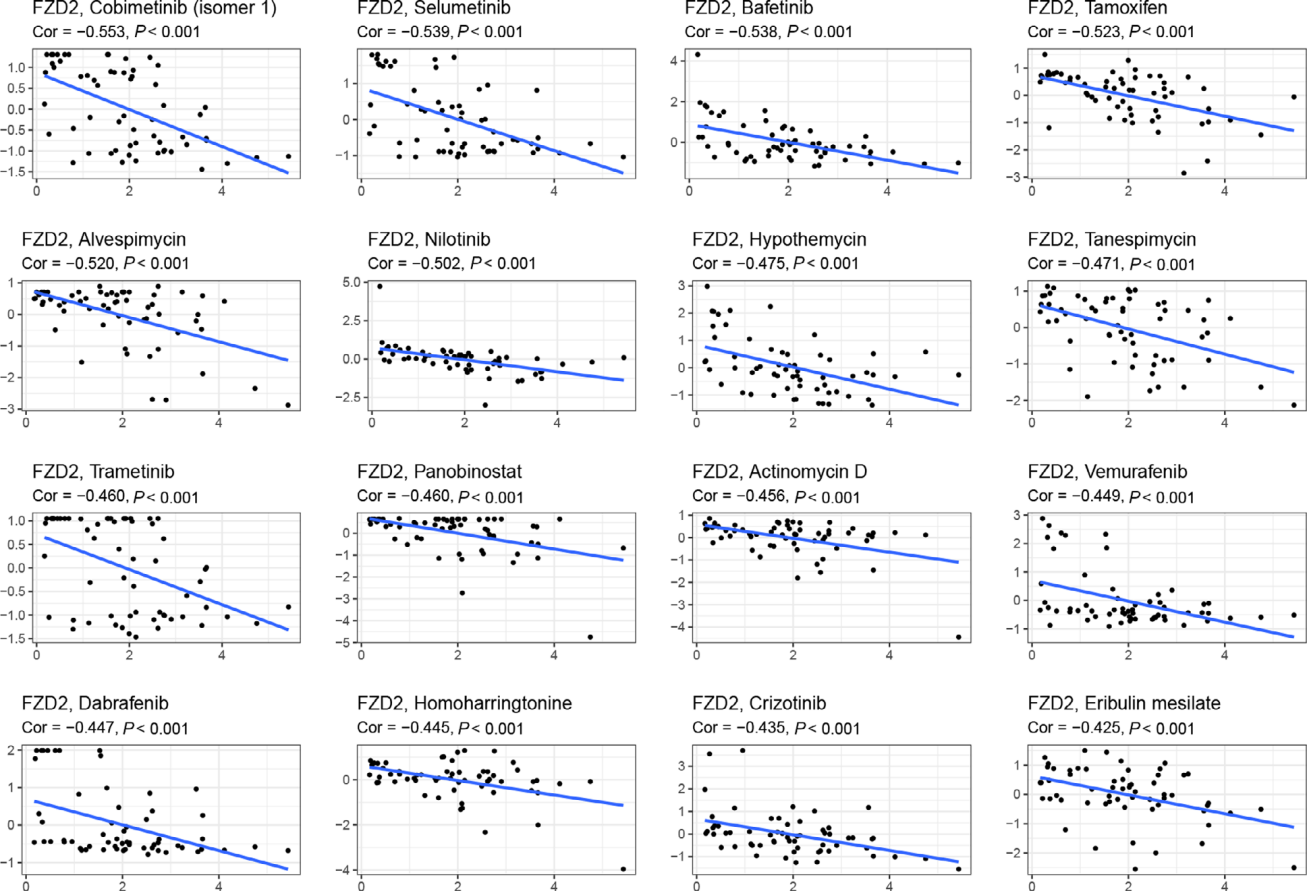
Drug response analysis. The correlation between drug sensitivity and *FZD2* across TCGA cancers. The scatter plots are ranked by *P*‐value. Spearman's correlation tests were used for testing, and *P* < 0.05 was considered significant.

*FZD2* was found to be related to a variety of drug sensitivities, including cobimetinib (*r* = −0.553, *P* < 0.001), selumetinib (*r* = −0.539, *P* < 0.001), bafetinib (*r* = −0.538, *P* < 0.001), tamoxifen (*r* = −0.523, *P* < 0.00 1), alvespimycin (*r* = −0.520, *P* < 0.001), and nilotinib (*r* = −0.502, *P* < 0.001), as well as other drugs that were closely related (Fig. 6). As the expression of *FZD2* increases, the cell sensitivity to drugs decreases.

### Related genes with FZD2 and their interacting protein network

**Fig. 7:**
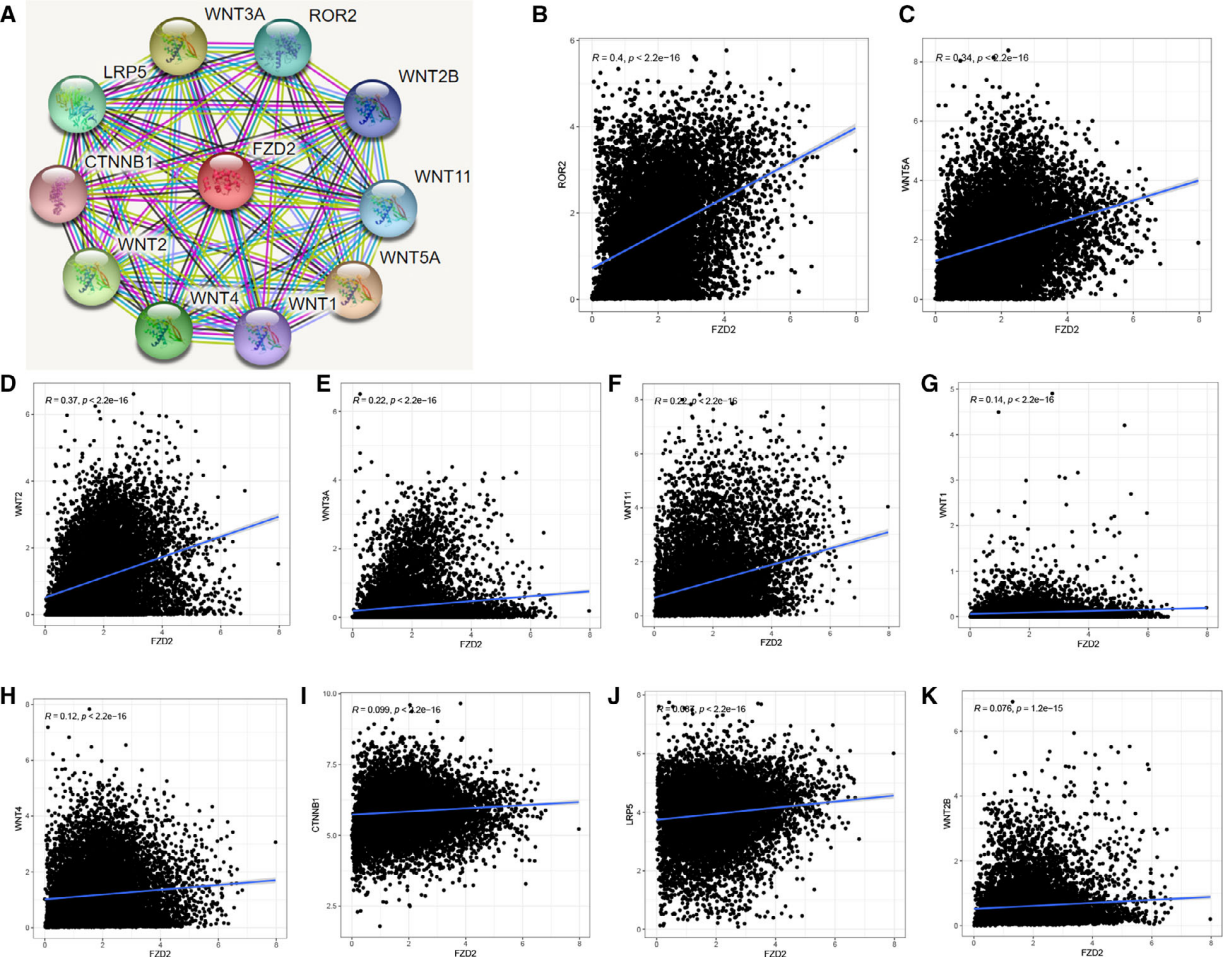
PPI network analysis. (A) The PPI network of FZD2 is constructed by STRING database. (B–K) Correlation analysis between FZD2 and main interacting genes in TCGA. Spearman's correlation tests were used for testing, and *P* < 0.05 was considered significant.

STRING was used to analyze the PPI with *FZD2* (Fig. 7A). The main interactions with *FZD2* in the PPI network were *LRP5*, *ROR2*, *Wnt2B*, *Wnt11*, *Wnt5A*, *Wnt1*, *Wnt4*, *Wnt2*, *CTNNB1*, and *Wnt3A*. Using the TCGA to analyze the correlation between *FZD2* and these genes (Fig. 7B–K), the results showed that *FZD2* had the most significant correlation with *ROR2* (*r* = 0.4, *P* < 0.001), *Wnt2* (*r* = 0.37, *P* < 0.001), and *Wnt4A* (*r* = 0.34, *P* < 0.001).

## Discussion

Abnormal activation of the Wnt signaling pathway causes abnormal accumulation of β‐catenin in tumor cells, leading to abnormal cell proliferation and tumor occurrence [3]. As the receptors of the Wnt signaling pathway, *FZDs* activate downstream signaling by binding to Wnt ligands, further regulating cell proliferation, differentiation, migration, tissue polarity, and tumor development. *FZDs* have been found to be specifically expressed on the cell plasma membrane, and *FZD2* is one of the most important receptors in the noncanonical Wnt pathway; it is highly expressed in many cancers and is a marker of poor prognosis [9, 19, 20].

Studies have found that the *FZD2* receptor protein can combine with *Wnt3A* activated by *ROR2* molecules to initiate the Wnt signaling classical pathway and act as a cancer‐promoting factor in lung cancer [21]. Similarly, our study found that *FZD2* has a significant correlation with *ROR2*. Gene expression profile analysis revealed that *FZD2* plays a key role in the occurrence of gastric cancer (GC) [22]. In addition, the latest research has found that *FZD2* is more highly expressed in hepatocellular carcinoma tissues than in adjacent tissues, and the recurrence‐free survival rate of patients with high *FZD2* expression is significantly lower than that of patients with low expression. Furthermore, *FZD2* expression is significantly correlated with the mesenchymal phenotype in HCC cell lines, and knocking out *FZD2* can inhibit the migration and invasiveness of liver cancer cells [23]. Studies have shown that *FZD2* can promote OSCC cell migration and invasion by regulating the *STAT3* pathway [24]. From the results of this study, according to the TCGA database, *FZD2* was highly expressed in a variety of cancers and was closely related to patient survival and clinical stage. Therefore, it was hypothesized that *FZD2* may act as an oncogene in most tumors.

Cancer stem cells (CSCs) are a small group of cells in tumors that have self‐renewal ability, strong tumor‐forming ability, and resistance to chemotherapy drugs and radiotherapy [25, 26]. They are the root of tumorigenesis, drug resistance, recurrence, and metastasis. The Wnt/β‐catenin signaling pathway regulates the self‐renewal of liver stem cells and liver CSCs [27, 28, 29, 30]. As the receptor of Wnt, it has been confirmed that some family members of *FZD* are related to tumor stem cells and drug resistance [31]; for example, FZD7 can regulate the function of stem cells in the stomach and intestinal epithelium, and *FZD7* expression increases in GC cells and tissues [32]. Knockout of *FZD7* or use of Wnt/β‐catenin inhibitors has been shown to reduce the stemness and chemoresistance of GC cells [33]. FZD8 is highly expressed in human lung cancer tissue samples and cell lines, and knockout of *FZD8* can increase the sensitivity of lung cancer cells to paclitaxel [34]. In addition, the analysis done in this study found that *FZD2* was significantly correlated with Wnt2 and Wnt4A. It appears that *FZD2* may affect the drug resistance of tumor cells through the Wnt signaling pathway.

Studies have shown that *FZD2* promotes migration and invasion of OSCC cells by regulating the *STAT3* pathway [24]. The *IL‐6*/ *STAT3* signaling pathway is related to the stemness of breast cancer cells [35, 36]; both cancer cells and stromal cells in the tumor microenvironment can produce *IL‐6*, which promotes breast cancer cell invasion, stemness, and drug resistance by activating *STAT3* [37]. This study also found that *FZD2* is associated with DNAss, RNAss, and stemness in some tumors, indicating that *FZD2* may play a role in stemness maintenance. Further analysis also found that *FZD2* is related to a variety of drug sensitivities, such as cobimetinib, selumetinib, bafetinib, tamoxifen, alvespimycin, and nilotinib. As the expression of *FZD2* increases, the sensitivity of cells to these drugs also decreases. This could mean that *FZD2* is related to chemotherapeutic drug resistance, and it regulates tumor cell stemness through the Wnt signaling pathway to cause drug resistance. These issues warrant further study for confirmation.

The Wnt pathway plays a vital role not only in cell development, survival, and proliferation, but also in immunity [38, 39]. DCs are antigen‐presenting cells that play an important role in the initiation and regulation of acquired immunity, and regulate the immune tolerance process. In the tumor microenvironment, Wnt binds to the co‐receptors *LRP5*/*LRP6* of the Wnt classic signaling pathway (expressed by DC cells), activates the classic Wnt signaling pathway, mediates immune tolerance, inhibits the immune response of effector T cells, and alters antitumor effects [40]. This study found that *FZD2* was significantly related to DCs and the tumor microenvironment in a variety of tumors. In addition, other studies have shown that Wnt1 molecules bind to the transmembrane receptor Frizzled, and co‐receptors *LRP5*/*LRP6* and *CD36* on the cell surface and upregulate the expression of *CD36* on macrophages by activating the classic Wnt signaling pathway and *PPAR‐γ* to promote macrophages. The function of these cells is to take up low‐density lipoproteins, thereby affecting the physiological activity of macrophages [41]. This study found that *FZD2* had a significant correlation with macrophage and T‐cell CD4+ in a variety of tumors. In addition, *FZD2* had a significant correlation with multiple immune checkpoints in various types of cancer. Further study is needed to determine whether *FZD2* affects the proliferation or drug resistance of tumor cells by affecting the tumor microenvironment or cellular immunity, and this conclusion needs to be further studied for confirmation.

Tumor mutation burden is an independent biomarker that has been discovered in a variety of tumor immunotherapies in recent years, and can be used to predict the efficacy of immunotherapy [42, 43]. Those with high TMB expression have been shown to benefit more from immune checkpoint inhibitor therapy [44]. TMB reflects the total number of replacement and insertion/deletion mutations per megabase in the exon coding region of the evaluated gene in the tumor cell genome. Driving gene mutations can lead to tumors, and a large number of somatic mutations can produce new antigens, which can activate T cells and cause immune responses [45]. Therefore, when the number of gene variants accumulates, more new antigens are produced, and there is a greater possibility of recognition by the immune system. Previous research in our group found that *FZD2* is related to tumor immunity. In this study, further analysis of the correlation between *FZD2*, TMB, and MSI was performed, and the results show that there is a link between *FZD2* expression and TMB and MSI in certain cancer types. Studies have shown that frameshift mutations of *AXIN2* and *TCF7L2* are common in GC with high MSI, and these mutations may promote the development of GC through the control of Wnt signaling [46]. MSI is now considered an indicator to distinguish the types of tumors in patients with COAD. It was also found that *FZD2* was mutated in breast, endometrial, large intestine, liver, lung, skin, and stomach cancer. In addition, the expression of *FZD2* was significantly correlated with MSI in BLCA, BRCA, COAD, KICH, LUSC, PAAD, PCPG, and STAD.

Although this study confirmed the involvement of *FZD2* in tumorigenesis, drug sensitivity, and tumor cell immunity, it does have some limitations. The data come entirely from open databases and have not been verified experimentally. Also, *FZD2* is highly expressed in a variety of tumors and is associated with poor prognosis, but despite this, the specific mechanism behind this action has not been verified. The expression of *FZD2* also has a certain correlation with drug sensitivity, tumor microenvironment, tumor immunity, TMB, and MSI, but there is lack of data confirming their correlation.

## Conclusions

*FZD2* was found to be highly expressed in various tumors, and this high expression is related to poor survival and disease progression. The expression of *FZD2* was also related to tumor drug sensitivity, tumor microenvironment, immune cell infiltration, immune checkpoint gene expression, and immunotherapy indicators (TMB, MSI). In summary, these results confirm the importance of *FZD2* expression in cancer prognosis and treatment and provide new clues for cancer treatment strategies.

## Conflict of interest

The authors declare no conflict of interest.

## Author contributions

MZ came up with the design and conception. MZ, XS, and YZ prepared material, collected data, and analyzed the data. MZ wrote the first draft of the manuscript. All authors commented on previous versions of the manuscript. All authors read and approved the final manuscript.

## Supporting information


**Fig. S1**. Pie chart showing the percentage of the different mutation types of *FZD2* in human cancers according to the COSMIC database.

**Fig. S2**. Correlation between *FZD2* mRNA expression levels and abundance of immune infiltrates in pan‐cancer from TIMER database.

## Data Availability

The datasets generated and/or analyzed during the current study are available in TCGA program (https://portal.gdc.cancer.gov). NCI‐60 cell line data are available at CellMiner (https://discover.nci.nih.gov/cellminer/home.do).
