# Supplementary material for: Analysis of the role of Frizzled 2 in different cancer types
Source: FEBS Open Bio. 2021 Feb 25;11(4):1195–208. doi: 10.1002/2211-5463.13111 (PMC8016138; doi:10.1002/2211-5463.13111)
Supplement: Supplementary file 1 — Fig. S1. Pie chart showing the percentage of the different mutation types of FZD2 in human cancers according to the COSMIC database. [file FEB4-11-1195-s002.pdf]

Supplementary Figure 1 Pie chart showing the percentage of the different mutation types of FZD2 in human cancers according to the COSMIC database.

Adrenal gland

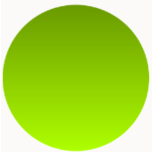

| Colour | Mutation type | Number of samples (%) |
|---|---|---|
| | Nonsense substitution | 0 (0.00%) |
| | Missense substitution | 1 (100.00%) |
| | Synonymous substitution | 0 (0.00%) |
| | Inframe insertion | 0 (0.00%) |
| | Frameshift insertion | 0 (0.00%) |
| | Inframe deletion | 0 (0.00%) |
| | Frameshift deletion | 0 (0.00%) |
| | Complex mutation | 0 (0.00%) |
| | Other | 0 (0.00%) |
| | Total unique samples | 1 |

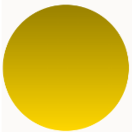

| Colour | Mutation type | Number of samples (%) |
|---|---|---|
| | A>C | 0 (0.00%) |
| | A>G | 0 (0.00%) |
| | A>T | 0 (0.00%) |
| | C>A | 1 (100.00%) |
| | C>T | 0 (0.00%) |
| | C>G | 0 (0.00%) |
| | G>A | 0 (0.00%) |
| | G>C | 0 (0.00%) |
| | G>T | 0 (0.00%) |
| | T>A | 0 (0.00%) |
| | T>C | 0 (0.00%) |
| | T>G | 0 (0.00%) |
| | Total unique samples | 1 |

Autonomic ganglia

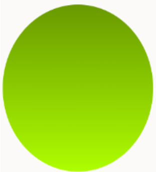

| Colour | Mutation type | Number of samples (%) |
|---|---|---|
| | Nonsense substitution | 0 (0.00%) |
| | Missense substitution | 1 (100.00%) |
| | Synonymous substitution | 0 (0.00%) |
| | Inframe insertion | 0 (0.00%) |
| | Frameshift insertion | 0 (0.00%) |
| | Inframe deletion | 0 (0.00%) |
| | Frameshift deletion | 0 (0.00%) |
| | Complex mutation | 0 (0.00%) |
| | Other | 0 (0.00%) |
| | Total unique samples | 1 |

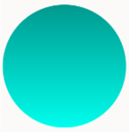

| Colour | Mutation type | Number of samples (%) |
|---|---|---|
| | A>C | 0 (0.00%) |
| | A>G | 0 (0.00%) |
| | A>T | 0 (0.00%) |
| | C>A | 0 (0.00%) |
| | C>T | 0 (0.00%) |
| | C>G | 1 (100.00%) |
| | G>A | 0 (0.00%) |
| | G>C | 0 (0.00%) |
| | G>T | 0 (0.00%) |
| | T>A | 0 (0.00%) |
| | T>C | 0 (0.00%) |
| | T>G | 0 (0.00%) |
| | Total unique samples | 1 |

Biliary tract

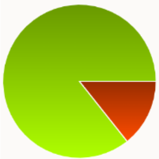

| Colour | Mutation type | Number of samples (%) |
|---|---|---|
| | Nonsense substitution | 0 (0.00%) |
| | Missense substitution | 6 (85.71%) |
| | Synonymous substitution | 1 (14.29%) |
| | Inframe insertion | 0 (0.00%) |
| | Frameshift insertion | 0 (0.00%) |
| | Inframe deletion | 0 (0.00%) |
| | Frameshift deletion | 0 (0.00%) |
| | Complex mutation | 0 (0.00%) |
| | Other | 0 (0.00%) |
| | Total unique samples | 7 |

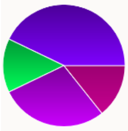

| Colour | Mutation type | Number of samples (%) |
|---|---|---|
| | A>C | 0 (0.00%) |
| | A>G | 0 (0.00%) |
| | A>T | 0 (0.00%) |
| | C>A | 0 (0.00%) |
| | C>T | 3 (42.86%) |
| | C>G | 0 (0.00%) |
| | G>A | 1 (14.29%) |
| | G>C | 0 (0.00%) |
| | G>T | 2 (28.57%) |
| | T>A | 0 (0.00%) |
| | T>C | 1 (14.29%) |
| | T>G | 0 (0.00%) |
| | Total unique samples | 7 |

Central nervous system

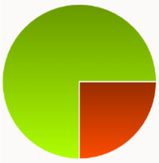

| Colour | Mutation type | Number of samples (%) |
|---|---|---|
| | Nonsense substitution | 0 (0.00%) |
| | Missense substitution | 3 (75.00%) |
| | Synonymous substitution | 1 (25.00%) |
| | Inframe insertion | 0 (0.00%) |
| | Frameshift insertion | 0 (0.00%) |
| | Inframe deletion | 0 (0.00%) |
| | Frameshift deletion | 0 (0.00%) |
| | Complex mutation | 0 (0.00%) |
| | Other | 0 (0.00%) |
| | Total unique samples | 4 |

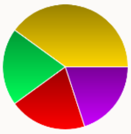

| Colour | Mutation type | Number of samples (%) |
|---|---|---|
| | A>C | 0 (0.00%) |
| | A>G | 0 (0.00%) |
| | A>T | 0 (0.00%) |
| | C>A | 2 (50.00%) |
| | C>T | 0 (0.00%) |
| | C>G | 0 (0.00%) |
| | G>A | 1 (25.00%) |
| | G>C | 1 (25.00%) |
| | G>T | 1 (25.00%) |
| | T>A | 0 (0.00%) |
| | T>C | 0 (0.00%) |
| | T>G | 0 (0.00%) |
| | Total unique samples | 4 |

# Cervix

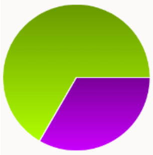

| Colour | Mutation type | Number of samples (%) |
|---|---|---|
| | Nonsense substitution | 0 (0.00%) |
| | Missense substitution | 2 (66.67%) |
| | Synonymous substitution | 0 (0.00%) |
| | Inframe insertion | 0 (0.00%) |
| | Frameshift insertion | 0 (0.00%) |
| | Inframe deletion | 0 (0.00%) |
| | Frameshift deletion | 0 (0.00%) |
| | Complex mutation | 0 (0.00%) |
| | Other | 1 (33.33%) |
| | Total unique samples | 3 |

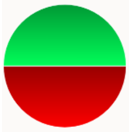

| Colour | Mutation type | Number of samples (%) |
|---|---|---|
| | A>C | 0 (0.00%) |
| | A>G | 0 (0.00%) |
| | A>T | 0 (0.00%) |
| | C>A | 0 (0.00%) |
| | C>T | 0 (0.00%) |
| | C>G | 0 (0.00%) |
| | G>A | 1 (50.00%) |
| | G>C | 1 (50.00%) |
| | G>T | 0 (0.00%) |
| | T>A | 0 (0.00%) |
| | T>C | 0 (0.00%) |
| | T>G | 0 (0.00%) |
| | Total unique samples | 2 |

# Haematopoietic and lymphoid

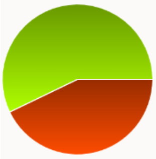

| Colour | Mutation type | Number of samples (%) |
|---|---|---|
| | Nonsense substitution | 0 (0.00%) |
| | Missense substitution | 4 (57.14%) |
| | Synonymous substitution | 3 (42.86%) |
| | Inframe insertion | 0 (0.00%) |
| | Frameshift insertion | 0 (0.00%) |
| | Inframe deletion | 0 (0.00%) |
| | Frameshift deletion | 0 (0.00%) |
| | Complex mutation | 0 (0.00%) |
| | Other | 0 (0.00%) |
| | Total unique samples | 7 |

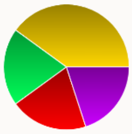

| Colour | Mutation type | Number of samples (%) |
|---|---|---|
| | A>C | 0 (0.00%) |
| | A>G | 0 (0.00%) |
| | A>T | 0 (0.00%) |
| | C>A | 2 (50.00%) |
| | C>T | 0 (0.00%) |
| | C>G | 0 (0.00%) |
| | G>A | 1 (25.00%) |
| | G>C | 1 (25.00%) |
| | G>T | 1 (25.00%) |
| | T>A | 0 (0.00%) |
| | T>C | 0 (0.00%) |
| | T>G | 0 (0.00%) |
| | Total unique samples | 4 |

# Kidney

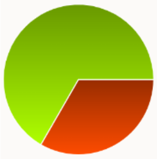

| Colour | Mutation type | Number of samples (%) |
|---|---|---|
| | Nonsense substitution | 0 (0.00%) |
| | Missense substitution | 4 (66.67%) |
| | Synonymous substitution | 2 (33.33%) |
| | Inframe insertion | 0 (0.00%) |
| | Frameshift insertion | 0 (0.00%) |
| | Inframe deletion | 0 (0.00%) |
| | Frameshift deletion | 0 (0.00%) |
| | Complex mutation | 0 (0.00%) |
| | Other | 0 (0.00%) |
| | Total unique samples | 6 |

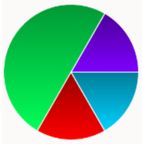

| Colour | Mutation type | Number of samples (%) |
|---|---|---|
| | A>C | 0 (0.00%) |
| | A>G | 0 (0.00%) |
| | A>T | 0 (0.00%) |
| | C>A | 0 (0.00%) |
| | C>T | 1 (16.67%) |
| | C>G | 0 (0.00%) |
| | G>A | 3 (50.00%) |
| | G>C | 1 (16.67%) |
| | G>T | 0 (0.00%) |
| | T>A | 1 (16.67%) |
| | T>C | 0 (0.00%) |
| | T>G | 0 (0.00%) |
| | Total unique samples | 6 |

# Liver

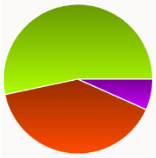

| Colour | Mutation type | Number of samples (%) |
|---|---|---|
| | Nonsense substitution | 0 (0.00%) |
| | Missense substitution | 8 (53.33%) |
| | Synonymous substitution | 6 (40.00%) |
| | Inframe insertion | 0 (0.00%) |
| | Frameshift insertion | 0 (0.00%) |
| | Inframe deletion | 0 (0.00%) |
| | Frameshift deletion | 0 (0.00%) |
| | Complex mutation | 0 (0.00%) |
| | Other | 1 (6.67%) |
| | Total unique samples | 15 |

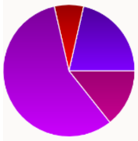

| Colour | Mutation type | Number of samples (%) |
|---|---|---|
| | A>C | 0 (0.00%) |
| | A>G | 0 (0.00%) |
| | A>T | 0 (0.00%) |
| | C>A | 0 (0.00%) |
| | C>T | 3 (21.43%) |
| | C>G | 0 (0.00%) |
| | G>A | 0 (0.00%) |
| | G>C | 1 (7.14%) |
| | G>T | 8 (57.14%) |
| | T>A | 0 (0.00%) |
| | T>C | 2 (14.29%) |
| | T>G | 0 (0.00%) |
| | Total unique samples | 14 |

## Meninges

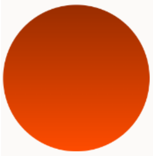

| Colour | Mutation type | Number of samples (%) |
|---|---|---|
| | Nonsense substitution | 0 (0.00%) |
| | Missense substitution | 0 (0.00%) |
| | Synonymous substitution | 1 (100.00%) |
| | Inframe insertion | 0 (0.00%) |
| | Frameshift insertion | 0 (0.00%) |
| | Inframe deletion | 0 (0.00%) |
| | Frameshift deletion | 0 (0.00%) |
| | Complex mutation | 0 (0.00%) |
| | Other | 0 (0.00%) |
| | Total unique samples | 1 |

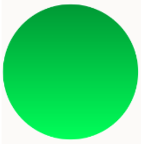

| Colour | Mutation type | Number of samples (%) |
|---|---|---|
| | A>C | 0 (0.00%) |
| | A>G | 0 (0.00%) |
| | A>T | 0 (0.00%) |
| | C>A | 0 (0.00%) |
| | C>T | 0 (0.00%) |
| | C>G | 0 (0.00%) |
| | G>A | 1 (100.00%) |
| | G>C | 0 (0.00%) |
| | G>T | 0 (0.00%) |
| | T>A | 0 (0.00%) |
| | T>C | 0 (0.00%) |
| | T>G | 0 (0.00%) |
| | Total unique samples | 1 |

## NS

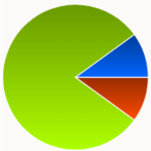

| Colour | Mutation type | Number of samples (%) |
|---|---|---|
| | Nonsense substitution | 1 (12.50%) |
| | Missense substitution | 8 (100.00%) |
| | Synonymous substitution | 1 (12.50%) |
| | Inframe insertion | 0 (0.00%) |
| | Frameshift insertion | 0 (0.00%) |
| | Inframe deletion | 0 (0.00%) |
| | Frameshift deletion | 0 (0.00%) |
| | Complex mutation | 0 (0.00%) |
| | Other | 0 (0.00%) |
| | Total unique samples | 8 |

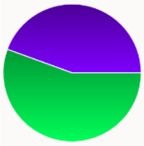

| Colour | Mutation type | Number of samples (%) |
|---|---|---|
| | A>C | 0 (0.00%) |
| | A>G | 0 (0.00%) |
| | A>T | 0 (0.00%) |
| | C>A | 0 (0.00%) |
| | C>T | 4 (50.00%) |
| | C>G | 0 (0.00%) |
| | G>A | 5 (62.50%) |
| | G>C | 0 (0.00%) |
| | G>T | 0 (0.00%) |
| | T>A | 0 (0.00%) |
| | T>C | 0 (0.00%) |
| | T>G | 0 (0.00%) |
| | Total unique samples | 8 |

## Oesophagus

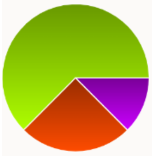

| Colour | Mutation type | Number of samples (%) |
|---|---|---|
| | Nonsense substitution | 0 (0.00%) |
| | Missense substitution | 5 (62.50%) |
| | Synonymous substitution | 2 (25.00%) |
| | Inframe insertion | 0 (0.00%) |
| | Frameshift insertion | 0 (0.00%) |
| | Inframe deletion | 0 (0.00%) |
| | Frameshift deletion | 0 (0.00%) |
| | Complex mutation | 0 (0.00%) |
| | Other | 1 (12.50%) |
| | Total unique samples | 8 |

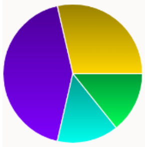

| Colour | Mutation type | Number of samples (%) |
|---|---|---|
| | A>C | 0 (0.00%) |
| | A>G | 0 (0.00%) |
| | A>T | 0 (0.00%) |
| | C>A | 2 (28.57%) |
| | C>T | 3 (42.86%) |
| | C>G | 1 (14.29%) |
| | G>A | 1 (14.29%) |
| | G>C | 0 (0.00%) |
| | G>T | 0 (0.00%) |
| | T>A | 0 (0.00%) |
| | T>C | 0 (0.00%) |
| | T>G | 0 (0.00%) |
| | Total unique samples | 7 |

## Ovary

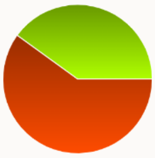

| Colour | Mutation type | Number of samples (%) |
|---|---|---|
| | Nonsense substitution | 0 (0.00%) |
| | Missense substitution | 2 (40.00%) |
| | Synonymous substitution | 3 (60.00%) |
| | Inframe insertion | 0 (0.00%) |
| | Frameshift insertion | 0 (0.00%) |
| | Inframe deletion | 0 (0.00%) |
| | Frameshift deletion | 0 (0.00%) |
| | Complex mutation | 0 (0.00%) |
| | Other | 0 (0.00%) |
| | Total unique samples | 5 |

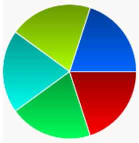

| Colour | Mutation type | Number of samples (%) |
|---|---|---|
| | A>C | 1 (20.00%) |
| | A>G | 1 (20.00%) |
| | A>T | 0 (0.00%) |
| | C>A | 0 (0.00%) |
| | C>T | 0 (0.00%) |
| | C>G | 1 (20.00%) |
| | G>A | 1 (20.00%) |
| | G>C | 1 (20.00%) |
| | G>T | 0 (0.00%) |
| | T>A | 0 (0.00%) |
| | T>C | 0 (0.00%) |
| | T>G | 0 (0.00%) |
| | Total unique samples | 5 |

## Pancreas

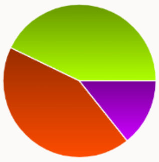

| Colour | Mutation type | Number of samples (%) |
|---|---|---|
| | Nonsense substitution | 0 (0.00%) |
| | Missense substitution | 3 (42.86%) |
| | Synonymous substitution | 3 (42.86%) |
| | Inframe insertion | 0 (0.00%) |
| | Frameshift insertion | 0 (0.00%) |
| | Inframe deletion | 0 (0.00%) |
| | Frameshift deletion | 0 (0.00%) |
| | Complex mutation | 0 (0.00%) |
| | Other | 1 (14.29%) |
| | Total unique samples | 7 |

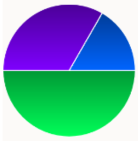

| Colour | Mutation type | Number of samples (%) |
|---|---|---|
| | A>C | 1 (16.67%) |
| | A>G | 0 (0.00%) |
| | A>T | 0 (0.00%) |
| | C>A | 0 (0.00%) |
| | C>T | 2 (33.33%) |
| | C>G | 0 (0.00%) |
| | G>A | 3 (50.00%) |
| | G>C | 0 (0.00%) |
| | G>T | 0 (0.00%) |
| | T>A | 0 (0.00%) |
| | T>C | 0 (0.00%) |
| | T>G | 0 (0.00%) |
| | Total unique samples | 6 |

## Prostate

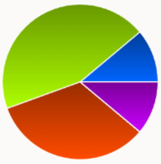

| Colour | Mutation type | Number of samples (%) |
|---|---|---|
| | Nonsense substitution | 1 (14.29%) |
| | Missense substitution | 4 (57.14%) |
| | Synonymous substitution | 3 (42.86%) |
| | Inframe insertion | 0 (0.00%) |
| | Frameshift insertion | 0 (0.00%) |
| | Inframe deletion | 0 (0.00%) |
| | Frameshift deletion | 0 (0.00%) |
| | Complex mutation | 0 (0.00%) |
| | Other | 1 (14.29%) |
| | Total unique samples | 7 |

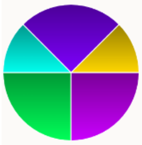

| Colour | Mutation type | Number of samples (%) |
|---|---|---|
| | A>C | 0 (0.00%) |
| | A>G | 0 (0.00%) |
| | A>T | 0 (0.00%) |
| | C>A | 1 (16.67%) |
| | C>T | 2 (33.33%) |
| | C>G | 1 (16.67%) |
| | G>A | 2 (33.33%) |
| | G>C | 0 (0.00%) |
| | G>T | 2 (33.33%) |
| | T>A | 0 (0.00%) |
| | T>C | 0 (0.00%) |
| | T>G | 0 (0.00%) |
| | Total unique samples | 6 |

## Small intestine

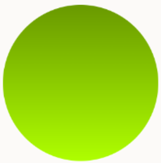

| Colour | Mutation type | Number of samples (%) |
|---|---|---|
| | Nonsense substitution | 0 (0.00%) |
| | Missense substitution | 1 (100.00%) |
| | Synonymous substitution | 0 (0.00%) |
| | Inframe insertion | 0 (0.00%) |
| | Frameshift insertion | 0 (0.00%) |
| | Inframe deletion | 0 (0.00%) |
| | Frameshift deletion | 0 (0.00%) |
| | Complex mutation | 0 (0.00%) |
| | Other | 0 (0.00%) |
| | Total unique samples | 1 |

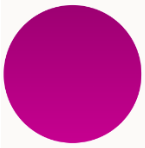

| Colour | Mutation type | Number of samples (%) |
|---|---|---|
| | A>C | 0 (0.00%) |
| | A>G | 0 (0.00%) |
| | A>T | 0 (0.00%) |
| | C>A | 0 (0.00%) |
| | C>T | 0 (0.00%) |
| | C>G | 0 (0.00%) |
| | G>A | 0 (0.00%) |
| | G>C | 0 (0.00%) |
| | G>T | 0 (0.00%) |
| | T>A | 0 (0.00%) |
| | T>C | 1 (100.00%) |
| | T>G | 0 (0.00%) |
| | Total unique samples | 1 |

## Soft tissue

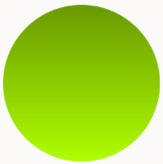

| Colour | Mutation type | Number of samples (%) |
|---|---|---|
| | Nonsense substitution | 0 (0.00%) |
| | Missense substitution | 1 (100.00%) |
| | Synonymous substitution | 0 (0.00%) |
| | Inframe insertion | 0 (0.00%) |
| | Frameshift insertion | 0 (0.00%) |
| | Inframe deletion | 0 (0.00%) |
| | Frameshift deletion | 0 (0.00%) |
| | Complex mutation | 0 (0.00%) |
| | Other | 0 (0.00%) |
| | Total unique samples | 1 |

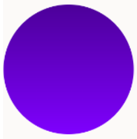

| Colour | Mutation type | Number of samples (%) |
|---|---|---|
| | A>C | 0 (0.00%) |
| | A>G | 0 (0.00%) |
| | A>T | 0 (0.00%) |
| | C>A | 0 (0.00%) |
| | C>T | 1 (100.00%) |
| | C>G | 0 (0.00%) |
| | G>A | 0 (0.00%) |
| | G>C | 0 (0.00%) |
| | G>T | 0 (0.00%) |
| | T>A | 0 (0.00%) |
| | T>C | 0 (0.00%) |
| | T>G | 0 (0.00%) |
| | Total unique samples | 1 |

## Testis

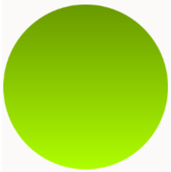

| Colour | Mutation type | Number of samples (%) |
|---|---|---|
| | Nonsense substitution | 0 (0.00%) |
| | Missense substitution | 1 (100.00%) |
| | Synonymous substitution | 0 (0.00%) |
| | Inframe insertion | 0 (0.00%) |
| | Frameshift insertion | 0 (0.00%) |
| | Inframe deletion | 0 (0.00%) |
| | Frameshift deletion | 0 (0.00%) |
| | Complex mutation | 0 (0.00%) |
| | Other | 0 (0.00%) |
| | Total unique samples | 1 |

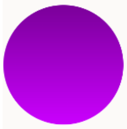

| Colour | Mutation type | Number of samples (%) |
|---|---|---|
| | A>C | 0 (0.00%) |
| | A>G | 0 (0.00%) |
| | A>T | 0 (0.00%) |
| | C>A | 0 (0.00%) |
| | C>T | 0 (0.00%) |
| | C>G | 0 (0.00%) |
| | G>A | 0 (0.00%) |
| | G>C | 0 (0.00%) |
| | G>T | 1 (100.00%) |
| | T>A | 0 (0.00%) |
| | T>C | 0 (0.00%) |
| | T>G | 0 (0.00%) |
| | Total unique samples | 1 |

## Thyroid

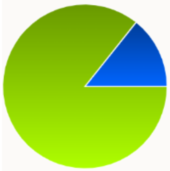

| Colour | Mutation type | Number of samples (%) |
|---|---|---|
| | Nonsense substitution | 1 (14.29%) |
| | Missense substitution | 6 (85.71%) |
| | Synonymous substitution | 0 (0.00%) |
| | Inframe insertion | 0 (0.00%) |
| | Frameshift insertion | 0 (0.00%) |
| | Inframe deletion | 0 (0.00%) |
| | Frameshift deletion | 0 (0.00%) |
| | Complex mutation | 0 (0.00%) |
| | Other | 0 (0.00%) |
| | Total unique samples | 7 |

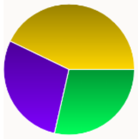

| Colour | Mutation type | Number of samples (%) |
|---|---|---|
| | A>C | 0 (0.00%) |
| | A>G | 0 (0.00%) |
| | A>T | 0 (0.00%) |
| | C>A | 3 (42.86%) |
| | C>T | 2 (28.57%) |
| | C>G | 0 (0.00%) |
| | G>A | 2 (28.57%) |
| | G>C | 0 (0.00%) |
| | G>T | 0 (0.00%) |
| | T>A | 0 (0.00%) |
| | T>C | 0 (0.00%) |
| | T>G | 0 (0.00%) |
| | Total unique samples | 7 |

## Upper aerodigestive tract

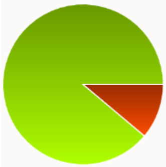

| Colour | Mutation type | Number of samples (%) |
|---|---|---|
| | Nonsense substitution | 0 (0.00%) |
| | Missense substitution | 8 (100.00%) |
| | Synonymous substitution | 1 (12.50%) |
| | Inframe insertion | 0 (0.00%) |
| | Frameshift insertion | 0 (0.00%) |
| | Inframe deletion | 0 (0.00%) |
| | Frameshift deletion | 0 (0.00%) |
| | Complex mutation | 0 (0.00%) |
| | Other | 0 (0.00%) |
| | Total unique samples | 8 |

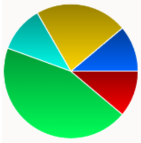

| Colour | Mutation type | Number of samples (%) |
|---|---|---|
| | A>C | 1 (12.50%) |
| | A>G | 0 (0.00%) |
| | A>T | 0 (0.00%) |
| | C>A | 2 (25.00%) |
| | C>T | 0 (0.00%) |
| | C>G | 1 (12.50%) |
| | G>A | 4 (50.00%) |
| | G>C | 1 (12.50%) |
| | G>T | 0 (0.00%) |
| | T>A | 0 (0.00%) |
| | T>C | 0 (0.00%) |
| | T>G | 0 (0.00%) |
| | Total unique samples | 8 |

## Urinary tract

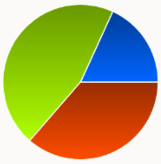

| Colour | Mutation type | Number of samples (%) |
|---|---|---|
| | Nonsense substitution | 2 (18.18%) |
| | Missense substitution | 5 (45.45%) |
| | Synonymous substitution | 4 (36.36%) |
| | Inframe insertion | 0 (0.00%) |
| | Frameshift insertion | 0 (0.00%) |
| | Inframe deletion | 0 (0.00%) |
| | Frameshift deletion | 0 (0.00%) |
| | Complex mutation | 0 (0.00%) |
| | Other | 0 (0.00%) |
| | Total unique samples | 11 |

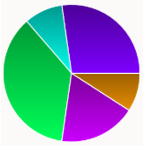

| Colour | Mutation type | Number of samples (%) |
|---|---|---|
| | A>C | 0 (0.00%) |
| | A>G | 0 (0.00%) |
| | A>T | 0 (0.00%) |
| | C>A | 0 (0.00%) |
| | C>T | 3 (27.27%) |
| | C>G | 1 (9.09%) |
| | G>A | 4 (36.36%) |
| | G>C | 0 (0.00%) |
| | G>T | 2 (18.18%) |
| | T>A | 0 (0.00%) |
| | T>C | 0 (0.00%) |
| | T>G | 1 (9.09%) |
| | Total unique samples | 11 |
