# Supplementary material for: Analysis of the role of Frizzled 2 in different cancer types
Source: FEBS Open Bio. 2021 Feb 25;11(4):1195–208. doi: 10.1002/2211-5463.13111 (PMC8016138; doi:10.1002/2211-5463.13111)
Supplement: Supplementary file 2 — Fig. S2. Correlation between FZD2 mRNA expression levels and abundance of immune infiltrates in pan‐cancer from TIMER database. [file FEB4-11-1195-s001.pdf]

Supplementary Figure 2. Correlation between FZD2 mRNA expression levels and abundance of immuneinfiltrates in pan-cancer from TIMER database.

	T cell CD8+	T cell CD4+	B cell	Neutrophil	Macrophage	Dendritic cell
ACC	-0.034	0.134	0.246	0.14	0.143	0.152
BLCA	0.226	-0.013	0.013	0.103	0.229	0.199
BRCA	0.03	0.022	-0.038	0.005	0.161	0.039
BRCA-Basal	0.012	-0.05	-0.075	0.09	0.082	0.027
BRCA-Her2	0.135	0.103	-0.224	0.226	0.289	0.376
BRCA-LumA	0.007	0.08	0.014	0.059	0.153	0.068
BRCA-LumB	-0.048	-0.021	-0.018	-0.039	0.006	0.03
CESC	-0.058	0.218	-0.001	-0.086	0.105	0.016
CHOL	0.096	0.047	0.042	-0.026	0.177	0.255
COAD	0.22	0.158	-0.006	0.48	0.257	0.509
DLBC	0.372	-0.038	-0.459	0.521	-0.468	0.485
ESCA	0.089	0.252	0.052	0.154	0.375	0.271
GBM	-0.075	0.113	-0.168	0.029	0.146	0.2
HNSC	-0.049	0.174	-0.15	0.184	0.364	0.23
HNSC-HPV-	-0.085	0.257	0.184	0.268	0.375	0.249
HNSC-HPV+	0.018	-0.059	0.009	-0.076	0.252	0.119
KICH	-0.105	0.172	-0.181	-0.07	0.066	0.074
KIRC	-0.08	0.183	-0.112	0.256	0.236	0.396
KIRP	0.134	0.229	0.161	0.183	0.096	0.201
LGG	-0.224	0.427	0.06	0.435	0.238	0.477
LIHC	0.141	0.471	0.452	0.301	0.44	0.663
LUAD	0.1	0.152	0.014	0.207	0.149	0.174
LUSC	0.124	0.197	0.03	-0.044	0.108	0.161
MESO	-0.016	0.115	0.14	-0.144	0.153	0.074
OV	-0.163	0.144	-0.325	-0.007	0.162	-0.259
PAAD	0.125	0.232	0.143	0.142	0.128	0.213
PCPG	-0.117	0.473	0.253	0.005	0.37	0.373
PRAD	-0.135	0.529	0.133	0.319	0.343	0.401
READ	0.09	0.302	0.019	0.233	0.368	0.349
SARC	0.105	-0.185	0.147	-0.051	0.076	-0.042
SKCM	-0.051	0.177	-0.089	0.142	0.161	0.105
SKCM-Metastasis	-0.054	0.136	-0.17	0.123	0.164	0.139

	T cell CD8+	T cell CD4+	B cell	Neutrophil	Macrophage	Dendritic cell
SKCM-Primary	-0.075	0.229	0.183	0.127	0.131	-0.119
STAD	0.267	0.21	0.013	0.126	0.389	0.248
TGCT	-0.449	0.215	-0.514	0.041	-0.046	-0.012
THCA	0.026	0.162	-0.056	-0.031	0.136	-0.028
THYM	-0.408	-0.101	-0.268	0.01	0.234	-0.22
UCEC	-0.206	0.088	-0.039	-0.035	-0.142	-0.063
UCS	0.02	0.065	-0.26	0.081	0.364	-0.105
UVM	0.05	0.019	-0.213	0.041	0.05	-0.013

Positive correlation($p<0.05$)

Negative correlation($p<0.05$)

Not significant($p>0.05$)
